# Comparative efficacy of pharmacotherapy and non‐pharmacotherapy for treating Alzheimer's disease: A systematic review and network meta‐analysis for cognitive, behavior and functional outcomes

**DOI:** 10.1002/alz70858_097093

**Published:** 2025-12-24

**Authors:** Chitima Boongird, Worapong Tearneukit, Wannisa Wongpipathpong, Unyaporn Suthutvoravut, Masatha Thongpan, Natthakorn Pongsettakul, Gareth J McKay, Sasivimol Rattanasiri, Thunyarat Anothaisintawee, Ammarin Thakkinstian

**Affiliations:** ^1^ Chakrinaruebodidra Institute, Ramathibodi Hospital, Mahidol University, Samutprakarn, Samutprakarn, Thailand; ^2^ Neuropsychiatry Unit, Somdet Chao Praya Institute of Psychiatry, Thailand, Bangkok, Bangkok, Thailand; ^3^ Department of Family Medicine, Ramathibodi Hospital, Mahidol University, Bangkok, Bangkok, Thailand; ^4^ Department of Psychiatry, Faculty of Medicine, Ramathibodi hospital, Mahidol University, Bangkok, Bangkok, Thailand; ^5^ Centre for Public Health, Queen's University Belfast, United Kingdom, Belfast, Belfast, United Kingdom; ^6^ Department of Clinical Epidemiology and Biostatistics, Faculty of Medicine Ramathibodi Hospital, Mahidol University, Bangkok, Bangkok, Thailand

## Abstract

**Importance:**

Alzheimer's disease (AD), the most prevalent form of dementia, contributes significantly to global disability. Pharmacotherapies (PTs) often have adverse effects, while diverse non‐pharmacotherapies (NPTs) lack comprehensive comparative evaluations against PTs.

**Objective:**

To assess the comparative efficacy of PTs and NPTs on cognitive, behavioral, and functional outcomes in AD.

**Data Sources:**

A systematic search was conducted in Medline and Scopus from inception to December 2022.

**Study Selection:**

Eligible studies included systematic reviews and randomized‐controlled trials (RCTs) that evaluated PTs, NPTs, or their combinations in AD patients with cognitive, behavioral, or functional outcomes.

**Data Extraction and Synthesis:**

Independent extraction was conducted by reviewer pairs, with discrepancies resolved by consensus. Risk of bias was assessed using the Revised Cochrane risk of bias tool (RoB 2.0). Fixed effect meta‐analysis was applied in the absence of heterogeneity, with a random effect model otherwise used. A two‐stage network meta‐analysis (NMA) enabled comparison of all interventions, with efficacy ranked using surface under the cumulative ranking curve (SUCRA).

**Main Outcomes and Measures:**

Cognitive, behavioral, and functional status.

**Results:**

A total of 153 RCTs were included. NMA findings indicated that, compared with placebo/usual care, donepezil combined with cognitive therapy (CT), rivastigmine with cognitive rehabilitation (CR), physical exercise (PE), CT, cognitive stimulation (CS), brain stimulation, donepezil, and huperzine significantly improved Mini‐Mental State Examination (MMSE) scores. Rivastigmine plus CS, brain stimulation with PE, PE alone, occupational therapy (OT), multicomponent interventions, donepezil with memantine, and Egb761 showed significant behavioral improvements. Only PE yielded significant functional status improvements. SUCRA rankings indicated rivastigmine with CR (93.3%), brain stimulation plus PE (93.1%), and rivastigmine with CS (86.8%) as the most effective for cognitive, behavioral, and functional outcomes, respectively.

**Conclusions and Relevance:**

Specific PT and NPT combinations, including donepezil+CT, rivastigmine+CR, and huperzine, showed notable cognitive benefits, while only PE significantly enhanced functional status. These findings suggest the potential for combined PT and NPT approaches in AD care; however, further research is needed to substantiate these results given the limited data on combination therapies.

